# Framing maternal morbidity: WHO scoping exercise

**DOI:** 10.1186/1471-2393-13-213

**Published:** 2013-11-19

**Authors:** Rachel C Vanderkruik, Özge Tunçalp, Doris Chou, Lale Say

**Affiliations:** 1 Department of Applied Research and Evaluation, National Initiative for Children’s Healthcare Quality (NICHQ), Boston, MA, USA; 2 UNDP/UNFPA/UNICEF/WHO/World Bank Special Programme of Research, Development and Research Training in Human Reproduction (HRP), Department of Reproductive Health and Research, World Health Organization, Geneva, Switzerland

## Abstract

**Background:**

Maternal morbidity estimations are not based on well-documented methodologies and thus have limited validity for informing efforts to address the issue and improve maternal health. To fill this gap, maternal morbidity needs to be clearly defined, driving the development of tools and indicators to measure and monitor maternal health. This article describes the scoping exercise conducted by the World Health Organization’s Department of Reproductive of Health and Research (WHO/RHR), as an essential first step in this process.

**Methods:**

A literature review was conducted to identify the range of definitions and conditions included in various studies of maternal morbidity with a special focus on the similarities and discrepancies of the definitions used across the studies. Furthermore a questionnaire was developed which included sections on key areas identified during the review and was sent out electronically to 130 international experts in the field of maternal health.

**Results:**

Maternal morbidities have been categorized in a variety of ways based on the causes, types of complications, and/or timeline. Issues regarding the time frame, severity, identification and classification and demographics were identified as key areas in the literature that require further investigation to achieve consensus on a maternal morbidity definition. Fifty-five (N = 55) individuals responded with completed questionnaires. Respondents’ views on the time frame for the postpartum period varied from 6 weeks to beyond one year postpartum, it was noted that time frame depended on the type of complication. The majority of respondents said maternal morbidity should comprise a continuum of severity, whereas the identification of the cases should use a mixed criteria employing multiple methods.

**Conclusions:**

Significant discrepancy in literature and expert opinion exists concerning elements of a maternal morbidity definition. There is a clear need for a concrete definition that would allow for consistent measurement and monitoring of maternal morbidity across settings and time.

## Background

Improving maternal health and reducing related mortality have been key concerns of the international community, especially as one of the eight Millennium Development Goals (MDG 5). However, to fully pursue MDG 5 and the goal of improving maternal health, it is important to broaden a focus to the entire spectrum of maternal morbidity, beyond maternal mortality. Complications of pregnancy, childbirth, and the postpartum period may lead to death or cause a continuum of morbidities that affect a woman’s health for short or long-term periods during and after pregnancy, and even throughout her life.

Community-based studies conducted in various countries have reported that women suffer significant morbidity both during pregnancy and in the postnatal period [1-4]. Such morbidities are also associated with poor fetal and newborn outcomes. Maternal morbidity is referred to as the base of the iceberg where maternal deaths are only the tip [5], and it has been suggested that for each maternal death, 20 or 30 women suffer from morbidity [6,7]. However, these calculations are not based on standard, well documented and transparent methodologies, thus have limited usefulness and validity for informing efforts to address the challenge of maternal morbidity.

Maternal mortality and maternal near miss have been defined by the World Health Organization, and there is a growing body of evidence from low and high resource settings on severe, life-threatening maternal morbidity. However, currently there is a lack of an agreed-upon definition for maternal morbidity. Existing work on maternal morbidity include an array of conditions, both short- and long-term in varying combinations [8-11]. Three major issues have limited valid, routine, and comparable measurements of maternal morbidity to date. These are: the lack of a common definition and identification criteria for maternal morbidity, lack of standardized assessment tools especially at community or primary health care level, and lack of common indicators to measure morbidity. To accurately monitor the improvement of maternal health, a definition for maternal morbidity is first needed, which will then drive development of tools and classifications to measure and monitor complete maternal health. The World Health Organization’s Department of Reproductive Health and Research (WHO/RHR) has convened a Maternal Morbidity Working Group that has embarked on a new project to improve the scientific basis for defining, estimating, and monitoring the magnitude of maternal morbidity [12]. The purpose of this project is to develop: a common definition and identification criteria for maternal morbidity that will then be used to provide estimates of the magnitude of maternal morbidity, a validated assessment tool for measuring maternal morbidity at community and primary health care levels, and a set of indicators of maternal morbidity measurement for program monitoring and evaluation purposes.

The aim of this manuscript is to describe the scoping exercise which was conducted as a first step in this project. There were three key objectives to the scoping exercise: 1) to explore how maternal morbidity is currently defined in the literature, 2) identify where there are discrepancies and gaps in current research, and 3) to determine the value of undertaking full systematic reviews relevant to maternal morbidity concepts. The scoping exercise had two components: 1) a literature review on maternal morbidity definitions and 2) the development and distribution of a questionnaire that was sent to experts in maternal health around the world. A scoping study can be used to rapidly map the key concepts underlying a research area that has not been comprehensively reviewed before [13], and the findings of this scoping exercise are being used to inform future work in the broader maternal morbidity project.

## Methods

From June through August 2011, a literature review was performed to identify and synthesize the range and type of conditions included in the literature published in English over the past 20 years on maternal morbidity. Our research questions were: how is maternal morbidity defined in existing literature, what conditions are included in definitions of maternal morbidity, and how are such conditions classified and identified? The key words for our literature review search included the following: *maternal morbidity, maternal complications, obstetric complications, obstetric morbidity, postpartum morbidity, perinatal morbidity,* and *pregnancy complications*. We searched through PubMed, WHO electronic databases (Index Medicus for the Eastern Mediterranean Region, African Index Medicus, Western Pacific Region Index Medicus), WHO Reproductive Health Library as well as GoogleScholar.

We used a charting approach to synthesize and interpret the data collected from the literature review. This technique involved the creation of a ‘data charting form’ using the database programme Excel where information about the studies and outcomes were recorded and organized according to key issues and themes relevant to maternal morbidity [14]. Attention was paid to the similarities and discrepancies of the maternal morbidity definitions across the studies. This charting of key themes from the literature identified discrepancies in 1) the timeframe within which a maternal morbidity occurs, 2) the severity of conditions considered to be a maternal morbidity 3) the way in which maternal morbidities are classified, and 4) the ways in which maternal morbidities are identified. The emergence of these themes supported the development of the scoping exercise questionnaire. The questionnaire was created for the purpose of assessing expert opinion on the identified gaps in research regarding maternal morbidity, and included questions on the timeframe, severity, identification, and classification of maternal morbidity (see Additional file 1).

A modified Delphi method was used among ten identified reproductive and maternal health experts within the WHO headquarters in Geneva, Switzerland to revise and finalize the questions within the questionnaire [15,16]. The questionnaire was pilot tested by the identified health experts within WHO, who provided feedback on the content and phrasing of the questions. There were two rounds of review based on this modified Delphi methodology. The finalized questionnaire was then sent out electronically to 130 experts in the field of maternal and reproductive health across all 6 WHO geographic regions in October 2011. These experts were identified from the distribution lists of WHO Reproductive Health and Research Department’s relevant products and collaborators in related WHO working groups, technical advisory groups and maternal focal points, as identified by countries participating in country consultations for maternal mortality [17].

Implied consent was obtained as the questionnaire data were collected using an optional online questionnaire tool which gathers data anonymously. This paper describes the results of an anonymous Delphi-like survey, requesting professional expertise on technical matters. Questions of personal and sensitive nature were not part of the survey. These are not considered research participant studies, and are thus considered exempt from review board approvals. The quantitative portions of the questionnaire were analyzed using descriptive statistics and measures of central tendency. Themes were derived from this scoping exercise using a thematic analysis of the qualitative data gathered from open-ended questions in the survey. The themes identified in the literature review and charting approach described above guided the thematic analysis of qualitative data collected.

## Results

Our literature review and charting of key themes identified significant discrepancy along the following areas regarding the maternal morbidity definition: time frame, severity, identification and classification. Our scoping survey included questions related to these key themes, and also assessed demographic characteristics. The results will be presented under these key areas, summarizing the literature review and including the feedback from the questionnaires. There were fifty-five respondents to the survey representing all of the WHO geographic regions with the exception of the South-East Asia region (response rate = 42.3%). A summary of data on the respondents is provided in Table 1.

**Table 1:** Background information on the respondents

**Overall**	Fifty-five (N = 55) individuals responded with completed questionnaires (response rate = 42.3%). The sex of respondents was split evenly between male (48%) and female (52%).
**Geographic distribution**	Of the 55 respondents, the distribution among the WHO geographic regions was as follows: African regions (n = 8), Region of the Americas (n = 13), Eastern Mediterranean Region (n =6), European Region (n = 16), South-East Asia Region (n = 0), Western Pacific Region (n =2), and not reported (n = 10).
**Organizations represented**	Respondents categorized their organization type as government/ministry (n = 25), an academic/research institution (n = 13), a medical/health organization (n = 11), a United Nations agency (n = 1), other (n = 5).
**Work classifications**	Respondents described their work as being research/evaluation (n = 13), statistics (n = 13), health/medical and/or service delivery (n = 12), or teaching/training (n = 10), policymaking (n = 9), program development/management (n = 8), reproductive health/family planning services (n = 6), advocacy (n = 3), health communication (n = 2), or other (n = 5).
	*Note: Respondents could select more than one option for their work*.

### Time frame for maternal morbidity

Our literature review revealed inconsistencies regarding the time frame within which a condition must occur to be considered a maternal morbidity, particularly around pre-pregnancy and postpartum. Several articles include pre-existing conditions (prior to a pregnancy) as a maternal morbidity if these conditions are aggravated by the pregnancy [18,19]. Other literature does not include the pre-pregnancy time in their definition of maternal morbidity, and only consider complications that arise during pregnancy, delivery, or the postpartum period [20].

The time frame considered relevant for a postpartum maternal morbidity also varied. Some literature considered morbidities that occurred within 42 days after giving birth, others included maternal problems up to 24 weeks postpartum, and some studies even examined maternal morbidities occurring anytime within the whole year after [8,21,22]. Often the time frame was not specifically stated or defined in literature, and time frames of “preconception” or “postpartum” were used without defining the length of time for those time periods. It has been indicated that maternal morbidities can be either acute or chronic, lasting from a range of months to years, and suggested that an ideal source for morbidity surveillance should include data on morbidity occurring during the entire year after delivery [21,23].

In line with the literature, nearly all respondents (95%) to the scoping exercise questionnaire indicated that maternal morbidity is relevant to pregnancy, labour and delivery, and the postpartum period. Only 36% of the respondents indicated that maternal morbidity is relevant to the pre-pregnancy time period. When asked whether a maternal morbidity can include a health condition that exists before a woman becomes pregnant (i.e. asthma, cardiac disease, diabetes), just over half of respondents (53%) felt that the answer is positive only if the condition worsens, or causes difficulty during pregnancy, delivery and postpartum.

The responses varied concerning the amount of time postpartum within which the onset of a complication must occur to become considered a postpartum maternal morbidity (ranging from 6 weeks to more than 1 year) and the majority of respondents selected “6 weeks” (58%) or “1 year” (27%). It was suggested that the time frame might depend on the specific condition or type of complication. The responses were divided between keeping the time frame consistent with maternal mortality definition (42 days) and modifying the time frame to include conditions as long as they can be linked to the pregnancy. To accurately capture and monitor maternal morbidities, a more cohesive viewpoint on the time frame within which relevant complications must occur should be established.

### Severity of maternal morbidity

Our review of current literature revealed that researchers have utilized maternal morbidity definitions of varying severities that range from less severe complications of pregnancy, and extend to near miss morbidity [19,24,25]. Maternal near miss has been defined as “a woman who nearly died but survived a complication that occurred during pregnancy, childbirth or within 42 days of termination of pregnancy” [26] but it is apparent that other, less severe, conditions are also critical when considering maternal health. Some studies on maternal morbidity include conditions that are potentially less severe but a discomfort for a pregnant woman, such as nausea, bed rest, or a car crash injury [25]. Other research focused on more severe conditions, such as those which utilize pregnancy-associated hospitalizations as an indicator of maternal morbidity [19,27].

The majority of respondents (91%) to the scoping exercise questionnaire felt that maternal morbidity could be either a temporary or a permanent condition. When asked about a range of severities that should be considered a maternal morbidity, two-thirds of respondents (66%) said that any perinatal condition, even if it does not require hospitalization or treatment, but may result in discomfort or dissatisfaction for the woman (e.g. excessive vomiting, nausea, oedema, depression) should be considered a maternal morbidity. However, contrasting suggestions were made that to be considered a maternal morbidity the condition must require medical intervention or treatment other than routine medication for expected side effects. It is evident that there is not a clear consensus among surveyed experts regarding the relevant severities of conditions for a maternal morbidity.

About three-quarters of respondents (76%) felt that maternal morbidity should comprise a spectrum or continuum of severity. However it was also suggested that the categories along the continuum must be easy for all health workers to objectively identify for reporting purposes. A continuum of severity has been proposed that progresses from normal/healthy pregnancy, to morbidity, to severe morbidity, to near miss, and finally to death [28]. It was noted by the respondents that there is no clear cut off point to distinguish between levels of severity for maternal morbidity. However, a majority of the respondents agreed that there should a scoring system based on several factors such as organ system failure, surgical intervention, extended intubation as well as quality of life measures. While the majority of recent research and attention has been around severe and life-threatening maternal morbidities (i.e. near miss), the definition of maternal morbidity should likely consist of a spectrum of severity with thoughtful consideration of how to distinguish cut-off points along the spectrum for various conditions.

### Identification and classification of maternal morbidity

Current literature includes a wide range of methods for identifying and classifying maternal morbidity. Maternal morbidities have been grouped under various categories such as direct obstetric morbidity, indirect obstetric morbidity and psychological obstetric morbidity [29] or obstetric complications, pre-existing medical conditions [30,31], or categories of obstetric morbidity that occur during pregnancy, during delivery, or after delivery [32]. Another way to categorize potentially life-threatening maternal conditions has been by type of complication, such as hemorrhagic complications, hypertensive disorders, or management indicators of severity [31]. Yet another example of the variety of categorization is the grouping of selected obstetric complications into “non-severe conditions”, “severe conditions”, and “procedures” [33].

In addition to the wide range of how maternal morbidity conditions are categorized, the methodology for detecting a maternal morbidity varied across studies as well, including interview-based diagnosis and hospital records (e.g. birth/hospital discharge data) [34,35]. A set of uniform diagnostic criteria and methodology of identification would allow for comparisons of maternal morbidity measures across settings. Severe maternal morbidity monitoring programs of various countries often use different methods of defining severe acute maternal morbidity (SAMM), including the International Statistical Classification of Diseases (ICD) and procedure codes, management and organ failure-based, and diagnosis-based criteria. For example, two key obstacles indicated in the capacity to monitor severe maternal morbidity in Australia have been the poor discrimination of severity of disease in coding categories of ICD and the lack of nationally agreed clinical and data definitions for particular morbid conditions to monitor maternal morbidity through the National Perinatal Data Collection (NPDC) [36].

Several studies identified in our literature review used pregnancy-associated hospitalizations as a proxy for maternal morbidity, whereas other literature argues that this methodology would not capture the full range of complications which might occur in the community and not reach a facility [19,27,37]. It has been suggested that the use of both diagnoses and procedures, and pregnancy and general diagnosis codes in all available fields for the admission, increases the likelihood of identifying morbidity in this study on measuring maternal morbidity in routinely collected health data [38].

A challenge of determining the frequencies of severe reproductive morbidities in high resource countries may be due to the different types of surveillance and reporting among various hospitals or countries [39]. In low resource countries, or in settings where diagnostic services are unavailable or underutilized, many women may have suffered from disease but never been diagnosed [34]. This could be due to lack of accessible services, not easily recognizable symptoms, or the particular condition may be so common in the community that its symptoms are considered the norm [34].

In our questionnaire, the majority of respondents (83%) felt that cases of maternal morbidity should be identified by “mixed criteria” based on a combination of clinical criteria related to a specific disease, intervention/treatment received, organ system dysfunction-based criteria, self-report, hospitalization and/or ICU admission. It was also noted that classification should employ the “direct, indirect, incidental” approach that is used in classifying maternal mortality. Respondents provided their perspectives on the challenges to measuring maternal morbidity, including the notion that perceptions of illness vary between cultures and the resistance that some women have to discussing personal matters. Such barriers and challenges should be considered and further explored when developing a systematic methodology for identification and measurement of maternal morbidity internationally.

### Demographics and maternal morbidity

Our literature review highlighted that certain demographic characteristics might place a woman at greater risk for experiencing a maternal morbidity. In the questionnaire, the main demographic characteristics that respondents felt are associated with an increased risk of a woman experiencing a maternal morbidity were age (92%), income level (89%), geographic location (89%), and education level (81%). The value in understanding the relationship between demographic characteristics and maternal morbidity is that it allows for targeted attention and services to the populations that need it most.

## Discussion

This paper describes the findings of our scoping exercise on maternal morbidity which included a literature review and survey of expert opinion. Our initial literature review was performed to obtain a sense of the range of definitions and conditions included in various studies of maternal morbidity, paying particular attention to the similarities and discrepancies of the definitions used across the studies. Key areas of interest identified regarding how maternal morbidity has been discussed in the literature were: time frame, severity, identification and classification and demographics. Based on the literature review findings, a questionnaire was developed and finalized using a modified Delphi Method and it was sent to a group of selected experts.

Overall our scoping exercise indicated that significant discrepancy in literature and expert opinion exists concerning the elements of a maternal morbidity definition. Therefore it also underlines the clear need for a concrete definition that would allow for consistent measurement and monitoring of maternal morbidity across settings and time. It will be a challenge to establish consensus on an ideal process for identification and classification of maternal morbidities that applies to both high and low resource countries, given the varying availability and use of reproductive health and diagnostic services. It should be noted that there has been significant progress in efforts to measuring and monitoring aspects of severe maternal morbidity such as near miss. The WHO recently published a multi-country survey in 29 countries using the WHO maternal near miss approach [40,41]. Furthermore, research using the WHO near miss criteria have been conducted in different parts of sub-Saharan Africa, suggesting that in low-resource settings further evaluation of the tool may be needed to accurately capture occurrence of maternal near miss [42-44]. While this body of work focused on severe maternal morbidity and near miss is critical, there is still a need for a more comprehensive assessment of maternal morbidities, which are non-severe or non-life threatening. This scoping exercise and following efforts of the WHO Maternal Morbidity Working Group is currently working to fill that gap.

In the questionnaire used for this scoping exercise, the respondents favoured classifying maternal morbidities into categories such as direct, indirect and incidental as used for maternal mortality. However, in practice, the interpretation of what clinical conditions should be included in which category has been inconsistent. These inconsistencies have compounded the difficulties in interpreting statistics on maternal mortality and in part led to the development of the WHO application of ICD-10 to deaths during pregnancy, childbirth, and the puerperium: ICD-MM [45]. The classification system ultimately proposed by the Maternal Morbidity Working Group will draw lessons from that endeavour and further contribute to the 11^th^ revision of the key standards for health conditions ICD-11 [46,47].

There are several limitations to this exercise which should be mentioned. The literature review conducted to inform the development of our scoping exercise questionnaire was not a systematic review. However, a purpose of this scoping exercise was to assist in determining what further systematic reviews related to maternal morbidity are needed. Additionally, the response rate for our questionnaire was under 50%, but there was representation from all but one of the WHO geographic regions. On the other hand, the results provided valuable feedback on identifying the gaps in knowledge and lack of consensus around how maternal morbidity is defined which will inform the next phases of work in the broader maternal morbidity project. While the focus of this scoping exercise was on maternal health, we recognize the critical link between neonatal health and the health of the mother. Certain neonatal conditions could be related to the condition of the mother, or to other causes (e.g. medical care) and the relationship between the monitoring of maternal and neonatal complications is an area that warrants further attention and study.

The results of this scoping exercise were presented at the first Maternal Morbidity Working Group meeting held in Geneva, Switzerland in April 2012, and helped to direct the discussion towards achieving consensus on a definition for maternal morbidity. Planned results of the WHO maternal morbidity project are expected to substantially contribute to improving maternal health through strengthening information for global and local decision-making in allocating resources and planning interventions to reduce maternal morbidity. The new definition of maternal morbidity will also be incorporated in the ICD-11 and related health problems, further enhancing the sustainability of the outputs of this effort. Only after this work is completed, can there be reliable monitoring of progress towards MDG 5 and beyond.

## Conclusions

Current calculations of maternal morbidity rates are not based on standard, well documented, and transparent methodologies. Rigorous and routine measurements of maternal morbidity are necessary to inform policy and program decisions and resource allocations that will also help reducing maternal deaths, and long-term suffering and disability. Improved systems of measurement will also allow for comparison of maternal morbidity burden across settings within and between countries. The scoping exercise highlighted that significant discrepancy in literature and expert opinion exists concerning elements of a maternal morbidity definition. There is a clear need for a concrete definition that would allow for consistent measurement and monitoring of maternal morbidity across settings and time.

## Competing interests

The authors declare that they have no competing interests.

## Authors’ contributions

LS wrote the grant (including design) that funded this project. RV conducted the research, analyzed the data, and wrote the first draft of the paper. DC, OT, and LS contributed to the design and conception of the study, provided feedback on results and interpretation of the data, and commented on drafts of the manuscript. All authors approved the final manuscript.

## Pre-publication history

The pre-publication history for this paper can be accessed here:

http://www.biomedcentral.com/1471-2393/13/213/prepub

## Supplementary Material

Additional file 1 Template of the Maternal Morbidity Scoping Exercise Survey. Click here for file
