# Supplementary material for: Framing maternal morbidity: WHO scoping exercise
Source: BMC Pregnancy Childbirth. 2013 Nov 19;13:213. doi: 10.1186/1471-2393-13-213 (PMC3840647; doi:10.1186/1471-2393-13-213)
Supplement: Additional file 1 — Template of the Maternal Morbidity Scoping Exercise Survey. [file 1471-2393-13-213-S1.pdf]

Maternal Morbidity Scoping Exercise Survey

Introduction:
Scoping Exercise on Maternal Morbidity

There is currently a lack of an agreed-upon definition of "maternal morbidity". Existing work on maternal morbidity include an array of conditions, both short- and long-term in varying combinations. WHO, Department of Reproductive Health and Research (RHR) including the Special Programme of Research, Development, and Research Training in Human Reproduction (HRP) is embarking on a project to improve and standardize the definition of maternal morbidity. An essential first step is to conduct a scoping exercise to identify and synthesize the range and types of conditions included in the maternal morbidity concept.

As an expert in the field of maternal health, we hope that you will help us with the first step of this project by completing the following questionnaire. We will use the information gathered from this scoping exercise to help direct the synthesis of a definition of maternal morbidity applicable to a variety of settings. It should take about 20 minutes to complete this form. Your feedback and thoughtful responses are of greatest importance to us.

Thank you very much for your time and participation.

* SECTION A

Please read the follow questions regarding "maternal morbidity" and check all of the corresponding responses that you feel apply.

1) Time Frame

Maternal morbidity is relevant to the following time periods: *Check All that Apply*

- ☐ Pre-conception
- ☐ Pregnancy
- ☐ Labour & Delivery
- ☐ Postpartum
- ☐ Other

If you selected "Other", please elaborate: [*open text*]

A maternal morbidity can include a health condition that exists before a woman becomes pregnant (i.e. asthma, cardiac disease, diabetes)

- ☑ Yes
- ☐ No
- ☐ Only if the condition worsens, or causes difficulty, during pregnancy/delivery/postpartum
- ☐ Other

If you selected "Other", please elaborate: [*open text*]

Within what amount of time postpartum must the onset of a complication occur to be considered a postpartum maternal morbidity?

- ☐ 6 weeks
- ☐ 6 months
- ☐ 1 year
- ☐ More than 1 year
- ☐ Other

If you selected "Other", please elaborate: [*open text*]

Other comments pertaining to the TIME FRAME for maternal morbidity: [*open text*]

2) Severity

Maternal morbidity can be:

- ☐ A temporary condition
- ☐ A permanent condition
- ☐ Both

☐ Other

If you selected "Other", please elaborate: [*open text*]

A condition of the following severities should be considered a maternal morbidity: Check all that apply

- ☐ Any perinatal condition, even if it does not require hospitalization or treatment, but may result in discomfort or dissatisfaction for the woman (i.e. excessive vomiting, nausea, oedema, depression)
- ☐ The condition requires medical intervention or treatment
- ☐ The condition puts the mother's and/or neonate's life at risk
- ☐ Other

If you selected "Other", please elaborate: [*open text*]

Should a definition of maternal morbidity comprise a spectrum/continuum of severity?

- ○ Yes
- ○ No
- ○ Other

If you selected "Other", please elaborate: [*open text*]

The continuum below has been presented in some literature (Geller et al., 2004): *Normal/Healthy Pregnancy -> Morbidity -> Severe Morbidity-> Near Miss -> Death*

- What factors do you feel distinguishes "Healthy Pregnancy" from "Morbidity"? [*open text*]

Referring to the same continuum, what factors do you feel distinguishes "Morbidity" from "Severe Morbidity"? [*open text*]

If you were to create a scale of severity for maternal morbidity with a range of scores, what factors would you include in determining the score? *Check all that Apply*

- ☐ Organ system failure
- ☐ Transfusion/Other procedure
- ☐ Extended intubation
- ☐ ICU admission
- ☐ Surgical intervention
- ☐ Other

If you selected "Other", please elaborate: [*open text*]

Other comments pertaining to the SEVERITY of maternal morbidity: [*open text*]

---

3) Identification & Classification

Do you feel that cases of maternal morbidity should be identified by: *Check all that apply*

- ☐ Clinical criteria related to a specific disease
- ☐ Intervention/treatment received (management criteria)
- ☐ Organ system dysfunction-based criteria
- ☐ Self-Report
- ☐ Hospitalization / ICU admission
- ☐ Mixed Criteria (use of more than one method)
- ☐ Other

If you selected "Other", please elaborate: [*open text*]

How would you define a "direct" maternal morbidity? [*open text*]

How would you define an "indirect" maternal morbidity? [*open text*]

Other comments pertaining to the IDENTIFICATION/CLASSIFICATION of maternal morbidity: [*open text*]

4) Demographics

Do you feel that any of the following characteristics is associated with an increased risk of a woman experiencing a maternal morbidity?

- ☐ Age
- ☐ Income Level
- ☐ Marital Status
- ☐ Race/Ethnicity
- ☐ Education Level
- ☐ Geographic Location
- ☐ Other

If you selected "Other", please elaborate: [*open text*]

Please elaborate on any of the demographics which you selected above: [*open text*]

SECTION B

Please indicate whether you feel the following conditions should be considered a "maternal morbidity" where:

"**YES**" = You feel the condition should be considered a maternal morbidity

"**NO**" = You feel the condition should not be considered a maternal morbidity

"**Unsure**" = You are not sure if the condition should be considered a maternal morbidity

PLEASE CONSIDER THE FOLLOWING CONDITIONS WITHIN THE TIMEFRAME RELEVANT TO MATERNAL MORBIDITY THAT YOU SPECIFIED IN SECTION A

Infections

Sexually Transmitted Infections (STIs)

	YES	NO	Unsure
Classify as Maternal Morbidity	○	○	○

Urinary/Genital Tract Infections

	YES	NO	Unsure
Classify as Maternal Morbidity	○	○	○

Endometritis

	YES	NO	Unsure
Classify as Maternal Morbidity	○	○	○

Malaria

	YES	NO	Unsure
Classify as Maternal Morbidity	○	○	○

Other puerperal infections

	YES	NO	Unsure
Classify as Maternal Morbidity	○	○	○

Other / Comments: [*open text*]

Abortion

Spontaneous

	YES	NO	Unsure

	YES	NO	Unsure
Classify as Maternal Morbidity	○	○	○

Induced

	YES	NO	Unsure
Classify as Maternal Morbidity	○	○	○

Abortion Complications

	YES	NO	Unsure
Classify as Maternal Morbidity	○	○	○

Stillbirth

	YES	NO	Unsure
Classify as Maternal Morbidity	○	○	○

Ectopic/Molar Pregnancy

	YES	NO	Unsure
Classify as Maternal Morbidity	○	○	○

Other / Comments: [*open text*]

Long-Term Complications

Obstetric Fistula

	YES	NO	Unsure
Classify as Maternal Morbidity	○	○	○

Genital Prolapse

	YES	NO	Unsure
Classify as Maternal Morbidity	◌	◌	◌

Infertility

	YES	NO	Unsure
Classify as Maternal Morbidity	◌	◌	◌

Incontinence

	YES	NO	Unsure
Classify as Maternal Morbidity	◌	◌	◌

Other / Comments: [*open text*]

Mental Health

Depression During Pregnancy

	YES	NO	Unsure
Classify as Maternal Morbidity	◌	◌	◌

Postpartum Depression

	YES	NO	Unsure
Classify as Maternal Morbidity	◌	◌	◌

Post-Traumatic Stress Disorder (PTSD)

	YES	NO	Unsure
Classify as Maternal Morbidity	◌	◌	◌

Anxiety

	YES	NO	Unsure
Classify as Maternal Morbidity	○	○	○

Attempted Suicide

	YES	NO	Unsure
Classify as Maternal Morbidity	○	○	○

Psychosis

	YES	NO	Unsure
Classify as Maternal Morbidity	○	○	○

Other / Comment: [*open text*]

Nutritional Status & Weight

Anaemia

	YES	NO	Unsure
Classify as Maternal Morbidity	○	○	○

Malnutrition

	YES	NO	Unsure
Classify as Maternal Morbidity	○	○	○

Excessive Weight Gain

	YES	NO	Unsure
Classify as Maternal Morbidity	○	○	○

Low Weight Gain

	YES	NO	Unsure
Classify as Maternal Morbidity	○	○	○

Obesity

	YES	NO	Unsure
Classify as Maternal Morbidity	○	○	○

Other / Comment: [*open text*]

Other

Obstructed Labor

	YES	NO	Unsure
Classify as Maternal Morbidity	○	○	○

Prolonged Labor

	YES	NO	Unsure
Classify as Maternal Morbidity	○	○	○

Pre-Term Delivery

	YES	NO	Unsure
Classify as Maternal Morbidity	○	○	○

Placental & Amniotic Cavity Disorders

	YES	NO	Unsure
Classify as Maternal	○	○	○

	YES	NO	Unsure
Morbidity			

C-Section

	YES	NO	Unsure
Classify as Maternal Morbidity	○	○	○

C-Section Complications

	YES	NO	Unsure
Classify as Maternal Morbidity	○	○	○

Anaesthesia Complications

	YES	NO	Unsure
Classify as Maternal Morbidity	○	○	○

FGM-Asscociated Complications

	YES	NO	Unsure
Classify as Maternal Morbidity	○	○	○

Disorders of Breast/Lactation(i.e. Mastitis)

	YES	NO	Unsure
Classify as Maternal Morbidity	○	○	○

Pre-Existing Medical Condition(i.e. chronic hypertension/cardiac disease/asthma/diabetes)

	YES	NO	Unsure
Classify as Maternal Morbidity	○	○	○

Injury (Not Related to Pregnancy)(i.e. car crash/ domestic violence during pregnancy)

	YES	NO	Unsure
Classify as Maternal Morbidity	○	○	○

Other / Comment

Last Page

Which of the following fetal and neonatal outcomes/assessments do you feel should be explored in the context of a maternal morbidity?

- ☐ Hypoglycemia
- ☐ Sepsis Evaluation
- ☐ Intravenous Fluids for Hypoglycemia
- ☐ Intrauterine growth restriction
- ☐ Macrosomia
- ☐ Low birth weight
- ☐ Fetal Anomalies
- ☐ Other

Other comments about any association between neonatal outcomes and maternal morbidity: [*open text*]

Which of the following conditions do you feel should have a defined threshold of severity to be considered a morbidity?

- ☐ Sexually Transmitted Infections (STIs)
- ☐ Urinary/Genital Tract Infections
- ☐ Endometritis
- ☐ Malaria
- ☐ Other puerperal Infections
- ☐ Spontaneous Abortion
- ☐ Induced Abortion
- ☐ Abortion Complications

- ☐ Stillbirth
- ☐ Ectopic/Molar Pregnancy
- ☐ Obstetric Fistula
- ☐ Genital Prolapse
- ☐ Infertility
- ☐ Incontinence
- ☐ Depression During Pregnancy
- ☐ Postpartum Depression
- ☐ PTSD
- ☐ Anxiety
- ☐ Attempted Suicide
- ☐ Psychosis
- ☐ Anaemia
- ☐ Malnutrition
- ☐ Excessive Weight Gain
- ☐ Low Weight Gain
- ☐ Obesity
- ☐ Obstructed Labor
- ☐ Prolonged Labor
- ☐ Pre-term Delilvery
- ☐ Placental & Amniotic Cavity Disorders
- ☐ C-Section
- ☐ C-Section Associated Complications
- ☐ FGM-Associated Complications
- ☐ Disorders of Breast/Lactation
- ☐ Pre-Existing Medical Condition
- ☐ Injury (Not-Related to Pregnancy)
- ☐ Anaesthesia Complications
- ☐ Other: ______

Final Comments

Please let us know if you feel anything not included in this questionnaire should be considered when working towards defining "maternal morbidity", or if you have any other comments/suggestions.

Information about Respondents to this Questionnaire

To help us understand who is responding to this questionnaire, please answer the following questions.

In which WHO region are you currently working?

- ☐ African Region
- ☐ Region of the Americas
- ☐ Eastern Mediterranean Region
- ☐ European Region
- ☐ South-East Asia Region
- ☐ Western Pacific Region

In what country are you currently working? ____

Please select the category that best describes your organization type: *Please choose ONE that is most applicable*

- ☐ Medical/Health Organization
- ☐ NGO/PVO (local and international)
- ☐ Government/Ministry
- ☐ Academic/Research institution
- ☐ Private sector (for profit)
- ☐ Faith-based organization
- ☐ United Nations agency
- ☐ Other: ____

Please select the category that best describes your work: *Please choose ONE that is most applicable*

- ☐ Advocacy
- ☐ Health communication
- ☐ Health/Medical and/or service delivery
- ☐ Reproductive Health/Family Planning Services
- ☐ Policymaking
- ☐ Program development/Management
- ☐ Research/Evaluation
- ☐ Teaching/Training
- ☐ Student
- ☐ Statistics
- ☐ Other: ____

What is your sex?

- ☐ Male
- ☐ Female

Again, thank you for your time and participation!
